# Implicit and explicit learning strategies and fatigue: an evaluation of throwing task performance

**DOI:** 10.3389/fpsyg.2025.1438313

**Published:** 2025-01-31

**Authors:** Reihaneh Banihosseini, Behrouz Abdoli, Maryam Kavyani

**Affiliations:** Faculty of Sport Sciences and Health, Department of Cognitive and Behavioral Sciences and Technology in Sport, Shahid Beheshti University of Tehran, Tehran, Iran

**Keywords:** implicit learning, explicit learning, working memory, mental fatigue, physical fatigue, errorless training, skill acquisition

## Abstract

**Introduction:**

This study aimed to determine the effects of implicit (errorless) and explicit (errorful) training strategies on a throwing task under physiological and mental fatigue conditions.

**Methods:**

Thirty-two participants, equally divided between the explicit and implicit learning groups, participated in a throwing task. The explicit learning group began at a significant distance from the target and gradually moved closer. In contrast, the implicit learning group started close to the target and progressively increased their distance. The initial session referred to as the acquisition phase, comprised 150 throws from five different distances. Subsequent sessions included a retention test and two transfer tests conducted under conditions of both physiological and mental fatigue. Mental fatigue was induced using a 30-minute color-word Stroop task, while physical fatigue was elicited by requiring subjects to maintain 50% of their maximum voluntary isometric contraction (MVC) in elbow extension for a 2-minute duration.

**Results:**

The results revealed that the implicit learning group exhibited improved performance under fatigue conditions and outperformed the explicit learning group significantly, regardless of the type of fatigue.

**Conclusion:**

This results suggests that implicit learning may improve motor performance even under fatigue conditions.

## 1 Introduction

Skills are fundamental to human life, guiding the pursuit of improved mental and physical learning for overall well-being. Extensive research is dedicated to unraveling the factors influencing motor skill learning. Central to this exploration is the debates surrounding explicit and implicit learning methods, with diverse factors scrutinized across various studies.

Implicit learning, as defined by Reber and Lewis (1977), involves knowledge acquisition independent of conscious effort and declarative knowledge. In contrast, explicit learning, as articulated by Masters and Maxwell (2004), engages conscious cognitive processes and declarative input. This dichotomy has been extensively investigated, revealing crucial differences, particularly concerning skill retention and transfer (Schmidt et al., 2018). Implicit learning, often characterized as non-conscious, operates autonomously of working memory, yielding procedural knowledge resistant to easy verbalization (Gentile, 1998; Maxwell et al., 2001). Working memory, a hypothesized cognitive structure, plays a critical role in processing verbal and visual information and detecting, correcting, and repairing performance errors (Davey et al., 2002; Berry and Dienes, 1993). Conversely, explicit learning relies on conscious, working memory-based processes for storing, retrieving, and processing declarative knowledge (Coutinho et al., 2018).

This study delves into the nuanced advantages of implicit learning methods, particularly focusing on errorless and errorful task training. Errorless training, aimed at reducing errors during learning, fortifies procedural and implicit memory (Gentile, 1998). In contrast, errorful learning, an explicit approach, relies on working memory to detect and correct errors (Maxwell et al., 2001).

The dynamic involvement of working memory and the impact of errors on skill acquisition are pivotal considerations in motor behavior. While variability in practice theory, rooted in schema theory (Baddeley and Hitch, 1974), underscores the benefits of varied task parameters and error recognition, reinvestment theory, proposed by Masters and Maxwell (2004), challenges this, advocating for implicit learning in fundamental skill acquisition (Baddeley, 1974; Cocchini et al., 2002; Masters and Maxwell, 2004).

Maxwell et al. (2001) demonstrated that minimizing errors and reducing working memory activity foster procedural and implicit learning. In another golf-related study by Maxwell et al. (2001), the errorless group, starting close to the hole, utilized implicit mechanisms, outperforming both the errorful and random groups (Maxwell et al., 2001; Savelsbergh et al., 2012).

The previous studies showed pervasive effect of physiological and mental fatigue on working memory, cognitive and motor performance that requires attention (Schmidt et al., 2018; Lam, 2008; Schmidt, 1975). Fatigue manifests as physical fatigue from prolonged tasks or mental fatigue stemming from extended cognitive engagement (Masters et al., 2008).

Studies on task performance under pressure underscore the resilience of implicitly learned processes, highlighting the coexistence of explicit and implicit cognitive pathways in motor performance (Masters and Maxwell, 2004; Hardy et al., 1996). Mental fatigue has been shown to impair selective attention, influencing tactical performance in sports (Faber et al., 2012; Badin et al., 2016). Moreover, cognitive fatigue has been associated with enhanced procedural sequence learning by imposing stress on working memory, emphasizing the potential of implicit memory resources (Borragán et al., 2016; Yang et al., 2020).

Borragán et al. (2016) demonstrated that cognitive fatigue, induced by stressing working memory, facilitates the learning of procedural sequences, relying on implicit memory resources(Borragán et al., 2016). In a recent review, Yang et al. (2020) compared implicit and explicit memory resources in motor skill automation, revealing superior motor skill automation in the implicit method (Yang et al., 2020). Consistent with this, cognitive fatigue has been found to potentially enhance procedural sequence learning (Borragán et al., 2016; Yang et al., 2020).

Observations on the influence of stress and fatigue in different learning groups, showing that the analogy and implicit groups were less affected by stress. In contrast, the explicit group showed a significant decrease in reaction time and accuracy (Lola et al., 2023). Furthermore, the analogy group demonstrated enhanced performance in improving selective attention in novices, both under normal and stressful conditions (Lola et al., 2023). These results show that cognitive overload, such as stress and fatigue, can have a significant negative effect on the accuracy and reaction time of the explicit group. In contrast it is unlikely to affect the implicit group.

This collective body of research underscores the crucial role of cognitive factors, such as stress and fatigue, in shaping implicit and explicit learning processes, impacting memory resources, procedural sequence learning, and overall task performance. The current study aims to contribute valuable insights by investigating the effects of errorless and errorful training under conditions of mental and physical fatigue.

In light of the literature review, we hypothesized that the implicit learning group would outperform in transfer tests under pressure, given the structural robustness of implicit memory compared to verbal memory under fatigue conditions. Our study seeks to fill a critical gap by examining the impact of errorless and errorful training methods under both types of fatigue. Through this research, we aim to unravel the nuanced differences in the effects of mental and physical fatigue on performance outcomes.

## 2 Materials and methods

### 2.1 Participants

Considering that the present study requires two groups and three measurement times, and that the researcher aims to achieve a minimum effect size of 0.3 according to the previous studies (Ramezanzade et al., 2022), a significance level of 0.05, and a statistical power of 0.95, it was estimated using G*Power software that a sample size of 32 participants would be sufficient to achieve these statistical levels with a 95% probability. Thirty-two participants, comprising 20 women and 12 men, with a mean age of (*M* ± SD = 29 ± 5.63), provided written informed consent to participate in the study. The Ethics Committee of the Faculty of Physical Education at Shahid Beheshti University approved the consent form used (Ethical code: IR.SBU.REC.1399.056). All participants had normal vision, were right-handed, had no history of psychiatric or motor disorders, and were novices at the throwing task of the study. In addition, participants who did not agree to continue or did not attend any of the three study sessions were excluded from the research process. To mitigate the impact of gender on results, participants were randomly assigned to two groups (errorful and errorless), each consisting of 10 women and 6 men. The Pittsburgh Sleep Quality Index indicated that all participants maintained normal sleep quality throughout the study period (*M* ± SD = 2 ± 1.41). A score of 6 or higher on this questionnaire indicates sleep disturbance (Buysse et al., 1989).

### 2.2 Throwing task

The task involved two target areas, requiring a tennis ball to impact the first target area on the ground before reaching the second. The first target area measured 60 × 60 in length × width was positioned 80 cm away from the second target area. The second target area was inclined at a 60-degree angle to the ground, featuring 10 concentric circles. Participants earned points (ranging from 1 to 10) based on their ball’s contact with each circle, starting from the largest to the smallest. No points were awarded if the ball failed to hit the first target before the second or if it did not touch the second target at all (Poolton et al., 2005; Singer, 1977). The entire recording and testing process was captured using a digital camera, and evaluation was conducted by reviewing the recorded footage.

### 2.3 Mental fatigue induction

Cognitive fatigue was induced using a thirty-minute word-color Stroop task. The duration and precise sequence of tests were established through preliminary testing with two participants before the main procedure (pilot study). To evaluate cognitive fatigue, a three-minute N-back test and a visual analog scale for fatigue assessment (VAS-F), were administered immediately before and after the Stroop task (Lee et al., 1991). The N-back test measured fatigue by examining the reduction in participants’ working memory capacity, as indicated by the decrease in their performance (percentage of correct answers) on the test (Lorist et al., 2000). A decrease of 20–30% in N-back test scores has been associated with indications of mental fatigue (Argüero Fonseca et al., 2023; Esposito et al., 2014).

### 2.4 Physical fatigue induction

Considering that a relatively limited group of arm muscles were involved in the throwing task and that the performance of throwing accuracy was directly affected by factors such as the strength and explosive power of the throwing hand, also based on the previous studies in this field, a local anaerobic protocol was used to induce physical fatigue (Poolton et al., 2007; Khalkhali et al., 2012).

Participants were tasked with sustaining an isometric contraction equivalent to 50% of their maximum voluntary contraction (MVC) in elbow extension for two minutes (Khalkhali et al., 2012). In this protocol, aimed at inducing anaerobic local fatigue in the throwing arm muscles, the triceps brachii is the primary muscle involved, followed by the biceps brachii and forearm muscles as the secondary muscles exhibiting the greatest activity. Additionally, shoulder muscles such as the deltoids and rotator cuff are activated to stabilize the position of the shoulder and arm in this posture (Colby, 2007; Wilmore and Costill, 1999). A manual dynamometer was utilized to maintain the isometric contraction. The participants’ MVC was assessed before and after the fatigue protocol, with an 18% decrease in MVC adopted as the fatigue criterion (Lepers et al., 2002). After the physical fatigue protocol, participants were prompted to use the Borg Scale (PRE), to measure their perceived exertion during the protocol (Borg, 1998).

## 3 Procedure

### 3.1 Pilot study

Before initiating the primary study protocol, a pilot study was conducted to validate the study tasks and familiarize the investigator with the procedural steps. This comprehensive pilot study included the entire main protocol and testing, involving two participants (one male, one female) who completed all three study sessions.

The pilot study aimed to determine the optimal duration of the Stroop color task for inducing mental fatigue. Participants reported experiencing fatigue and an inability to continue the test after 25 to 30 min, as indicated by a self-report fatigue scale. Consequently, the duration of the Stroop test for the main study was set at 30 min, based on these findings and in line with previous studies (Badin et al., 2016; Gonzalez et al., 2024; Cuchna et al., 2024).

### 3.2 Main study

No pretest was conducted in this study, as performing a pretest at distances less than 2.5 meters could result in a ceiling effect on power measurement. In such a scenario, all participants would start their throws from a short distance to the target. Moreover, a pretest with large distances to the target would undermine the effects of the errorless group, potentially leading to the formation of explicit knowledge in participants. Since all participants were amateurs with no prior task experience, a pretest was deemed unnecessary.

The acquisition session comprised 5 blocks of 30 trials each, with 50 s of rest between blocks. Before the acquisition session, 5 warm-up trials were conducted. The errorful learning group started from 3.5 meters away from the target, moving on to 3.25, 3, 2.75, and 2.5 meters from the target in consecutive blocks. The errorless learning group started from 2.5 meters away and moved on to 2.75, 3, 3.25, and 3.5 meters as the sessions progressed.

In the second session, two tests were administered 48 h after the acquisition session. The first test involved participants performing a single block of 10 trials from a distance of 3 meters as their retention test. Subsequently, after inducing mental fatigue through a Stroop test, participants performed another block of 10 trials from 3 meters away, serving as the first transfer test under mental fatigue. On the third day, 48 h after the second day, physical fatigue was induced, followed by a second transfer test (under physical fatigue) with a single block of 10 trials from 3 meters away.

**Figure 1:**
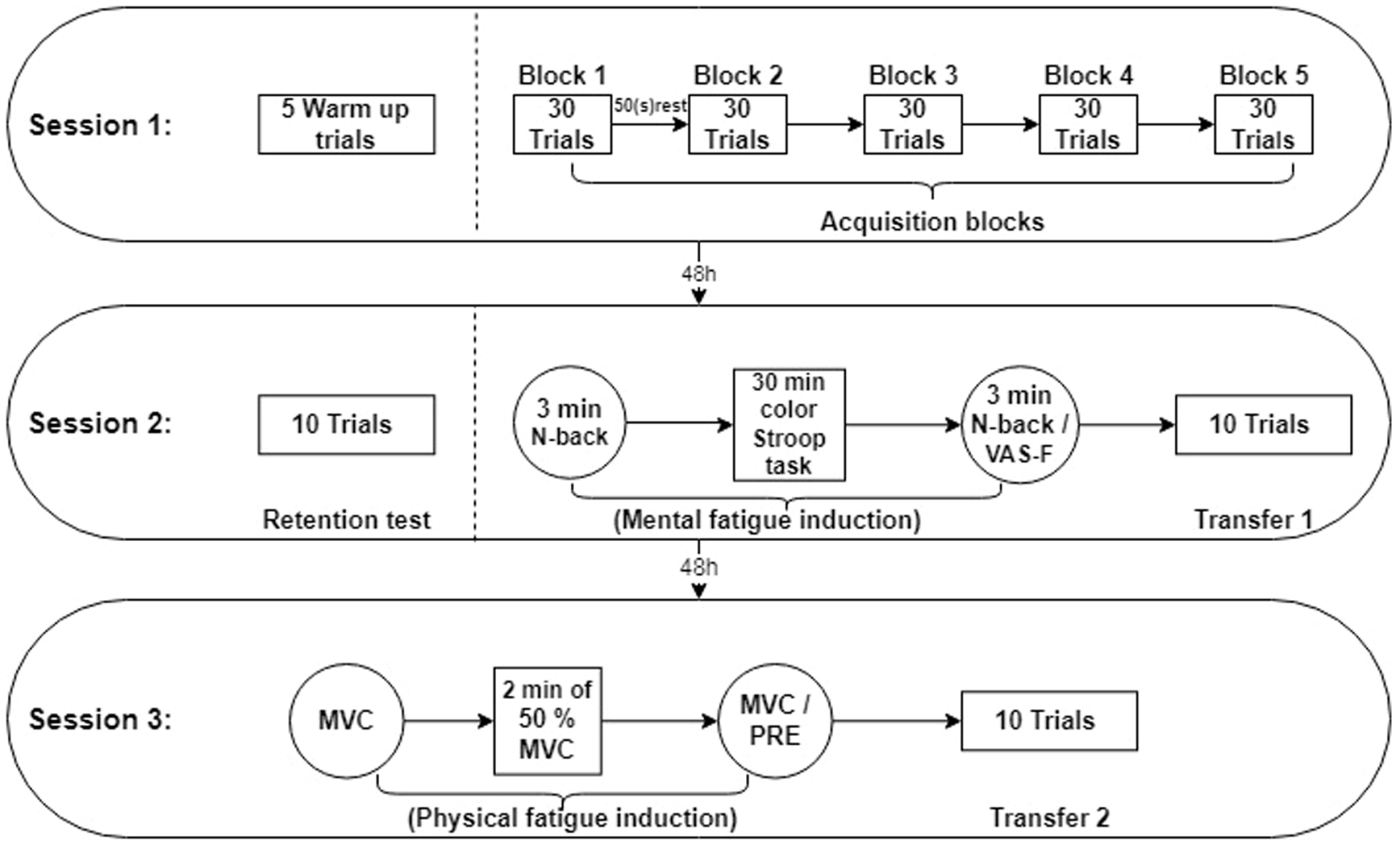
Exprimental Design. On the session 1, participants in each group completed blocks of 30 trials in throwing task. 48 h later, on the session 2 after the retention test, participants entered the transfer1 test (cognitive fatigue conditions). After 48 h on session3, the participants passed the transfer 2 test (physical fatigue conditions).

The entire study protocol, as illustrated in Figure 1, was subjected to statistical analysis. Scores, recorded by a digital camera, were calculated according to the point at which the ball hit the target. The Shapiro–Wilk test was used to assess the normality of the data, and the Leven’s test was used to test for homogeneity. To investigate the effects of time, group, and the interaction between time and group, a mixed ANOVA was employed. Subsequently, to further examine the differences between groups and the within-group changes, the Bonferroni *post-hoc* test was utilized. All statistical operations were conducted using IBM SPSS Statistics 27 software.

## 4 Results

Prior to conducting the mixed ANOVA, assumptions of normality were assessed using the Shapiro–Wilk test, which confirmed that the data were normally distributed across all groups and time points (*p* > 0.05). Homogeneity of variances was also verified using Levene’s test, ensuring that the mixed ANOVA assumptions were met.

### 4.1 Objective and subjective ratings of fatigue

**Figure 2:**
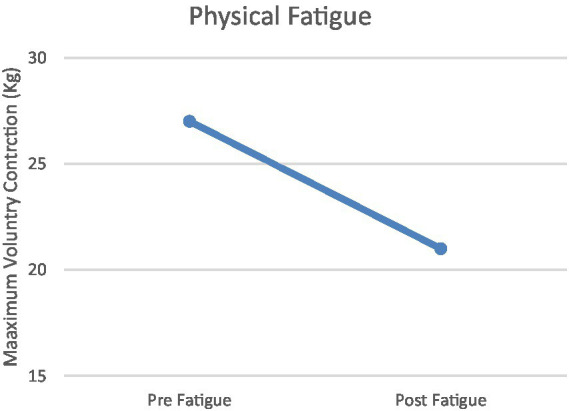
Physical Fatigue. Task-related physical fatigue (difference between the amount of MVC before and after the physical fatigue conditions) in participants.

**Figure 3:**
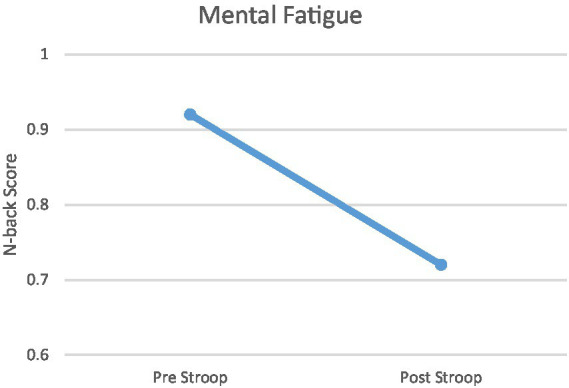
Mental Fatigue. Task-related mental fatigue (difference between N-back scores before and after the Stroop task) in participants.

Changes in MVC (maximal voluntary contraction) and N-back responses to physiological and mental fatigue states are shown in Figures 2, 3. The physical fatigue task resulted in a significant decrease (>18%) in MVC values after treatment compared to pretreatment in all participants (mean decrease = 21.01%, Figure 2). The mental fatigue task resulted in a significant decrease (>20%) in N-back scores after Stroop task compared to pretreatment in all participants (mean decease = 21.74%, Figure 3) Conversely, the BORG scale results, indicating perceived exertion rate, were significant after the physical fatigue treatment (*M* ± SD = 19.21 ± 1.38). The VAS scale (visual analog scale of fatigue) demonstrated significant effects of the mental fatigue protocol (*M* ± SD = 110 ± 10.38).

### 4.2 Acquisition phase

**Figure 4:**
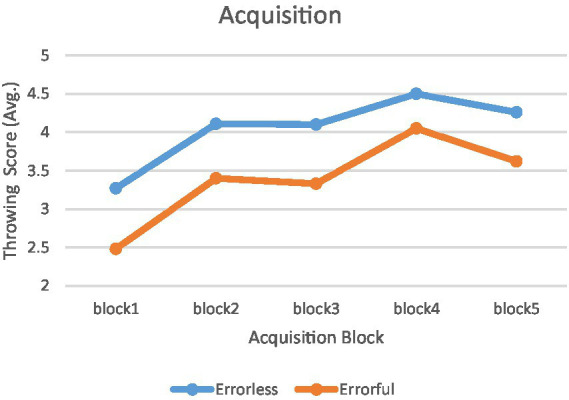
Acquisition phase. Mean performance accuracy for the errorless and errorful groups in the acquisition phase in 5 blocks.

The mixed ANOVA results indicate a significant main effect of time (F4,120 = 30.420, *p* < 0.001, *η*^2^ = 0.503) and a significant main effect of group (F1,30 = 18.968, *p* < 0.001, *η*^2^ = 0.387) on throwing scores. However, the interaction effect between group and time was not significant (F4, 120 = 0.509, *p* = 0.729, *η*^2^ = 0.017). Pair-wise comparisons using Bonferroni’s post-hoc analysis indicated that performance was consistently higher in the errorless group throughout the acquisition phase (*p* < 0.05). Furthermore, both groups exhibited a significant improvement from block 1 to block 5 (*p* < 0.05). The performance trajectories of the two test groups during the acquisition phase are shown in Figure 4.

### 4.3 Retention and transfers phase

The mixed ANOVA results revealed a significant interaction effect between group (errorless and errorful) and time (retention, transfer 1, and transfer 2) for throwing scores (F2, 60 = 154.148, *p* < 0.001, *η*^2^ = 0.837), indicating that the interventions had different impacts on the two groups over time. Additionally, a significant main effect of time was observed (F2, 60 = 3.688, *p* = 0.031, *η*^2^ = 0.109), suggesting that throwing performance changed significantly across the study phases regardless of group. The group effect was also significant (F1, 30 = 12.876, *p* = 0.001, *η*^2^ = 0.300), demonstrating overall differences in performance between the two groups (Table 1).

**Table 1:** Results of mixed ANOVA and Mean (SD) for throwing scores.

Variable	Group	Retention ^‡^	Transfer 1^‡^	Transfer 2^‡^	Interaction effect	Test time effect	Group effect
Throwing Score	Error less	2.03 ± 0.49	4.24 ± 0.67	4.58 ± 0.81	*F* = 154.148 *P* < 0.001* η2 = 0.837	*F* = 3.688 *p* = 0.031* η2 = 0.109	*F* = 12.876 *P* = 0.001* η2 = 0.300
	Error full	4.51 ± 0.65	2.07 ± 0.64	2.53 ± 0.73			

*Indicates significant *p*-value (*P* < 0.05).

^‡^Indicates significant between group difference (*p* < 0.05).

Based on the mixed ANOVA results, Bonferroni *post hoc* tests were conducted to compare between-group differences at each study phase. The post hoc analysis revealed that in the retention phase, the errorless group scored significantly lower (*M* = 2.03 ± 0.49) than the errorful group (*M* = 4.51 ± 0.65, *p* < 0.05) (Table 1).

In the first transfer phase, the errorless group scored significantly higher (*M* = 4.24 ± 0.67) compared to the errorful group (*M* = 2.07 ± 0.64, *p* < 0.05). This difference persisted in the second transfer phase, where the errorless group again showed significantly higher scores (*M* = 4.58 ± 0.81) compared to the errorful group (*M* = 2.53 ± 0.73, *p* < 0.05) (Table 1).

To examine within-group changes, Bonferroni post hoc tests were again used. The results revealed the following:

For the “errorless” group, the analysis showed a significant improvement in throwing performance from the retention phase to the first transfer phase (Mean Difference = −2.212, *p* < 0.001), with a Cohen’s d of-2.155, indicating a large effect size. This suggests a substantial improvement in performance between these two phases. A significant change was also observed from the retention phase to the second transfer phase (Mean Difference = −2.553, *p* < 0.001), with a Cohen’s d of-2.176, further confirming a large effect size. However, the difference between the first and second transfer phases was not significant (Mean Difference = −0.341, *p* = 0.205), with a Cohen’s d of-0.345, indicating a small effect size and suggesting that the change between these two phases was minimal.

**Figure 5:**
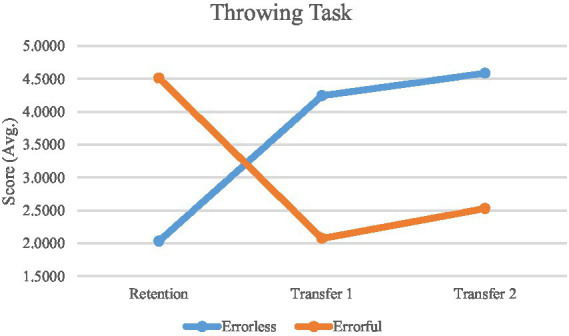
Performance comparison. Mean performance accuracy for the errorless and errorful groups in different phases of study (retention, transfer 1, transfer 2).

For the “errorful” group, there was a significant decline in throwing performance from the retention phase to the first transfer phase (Mean Difference = 2.439, *p* < 0.001), with a Cohen’s d of 3.565, indicating a very large effect size. This suggests a substantial decline in performance from the retention phase to the first transfer phase. Similarly, a significant decline was observed from the retention phase to the second transfer phase (Mean Difference = 1.983, *p* < 0.001), with a Cohen’s d of 2.968, indicating a large effect size. The difference between the first and second transfer phases approached significance (Mean Difference = −0.456, *p* = 0.051), with a Cohen’s d of-1.813, reflecting a large effect size but indicating that this difference was less consistent or reliable compared to the other comparisons (Table 2) (Figure 5).

**Table 2:** Bonferroni *post-hoc* test results for within-group changes.

Variable	Group	Phase	Phase	Mean difference	SIG	Cohen’s d
Throwing Score	Errorless	Retention	Transfer 1	−2.212	<0.001*	−2.155
			Transfer 2	−2.553	<0.001*	−2.176
		Transfer 1	Transfer 2	−0.341	=0.205	−0.345
	Errorful	Retention	Transfer 1	2.439	<0.001*	3.565
			Transfer 2	1.983	<0.001*	2.968
		Transfer 1	Transfer 2	−0.456	=0.051	−1.813

## 5 Discussion

The present study investigated the impact of implicit and explicit learning on motor task performance across three sessions. During the acquisition session, participants, divided into errorless (implicit) and errorful (explicit) groups, completed 150 throws from varying distances to the target. Subsequent sessions involved a retention test and the first transfer test (under mental fatigue) after 48 h, followed by the second transfer test conducted under physical fatigue conditions. The objective and subjective measures collectively provided comprehensive insights into the impact of physiological and mental fatigue on both performance and perceived exertion. Notably, the errorless group consistently outperformed the errorful group across sessions, demonstrating superior performance under both mental and physical fatigue conditions and even outperforming their retention tests. These findings align with previous research by Poolton et al. (2007), emphasizing the advantages of errorless training in reducing hypothesis testing and enhancing performance (Poolton et al., 2007).

The explicit group exhibited improved performance in the retention test, consistent with previous studies (Lola et al., 2023; Masters, 1992). This improvement is attributed to the explicit method facilitating the recognition of key environmental cues for optimal response analysis. The “reinvestment theory” suggests that under stress, explicit learners attempt to recall declarative knowledge, leading to regression in performance and increased cognitive load. Under both mental and physical fatigue conditions, the errorless group’s performance remained unaffected and even improved, in contrast to the explicit group, whose scores declined. This underscores the robustness of implicit memory, consistent with the literature on cognitive demands and implicit memory resources. Our results also align with Borragán et al. (2016), indicating that reducing working memory load during fatigue benefits implicit memory (Borragán et al., 2016). Following the confirmation that an increase in working memory load leads to improved performance in implicit memory, Masters et al. (2008) examined two conditions in a study on learning a surgical skill: explicit (instructions and observation) and implicit (observation only). They concluded that when simulating real-life situations and performing several tasks simultaneously, the implicit group showed more stable performance (Masters et al., 2008). The improved performance of the errorless group under fatigue conditions further supports the competitive relationship between working memory and implicit memory, favoring the latter under reduced cognitive resources (Banihosseini et al., 2023). In the study by Foerde et al. (2006), fMRI data showed that during the performance of probe tasks learned under dual-task conditions, there was increased activity in the putamen and striatal regions. Conversely, the right hippocampus and medial temporal lobe (MTL) activated more during tasks learned under single-task conditions (Foerde et al., 2006). This suggests a Competitive relationship between different memory systems (e.g., working memory and implicit memory) at the response level.

Furthermore, although these memory systems are separate, they collaborate on a similar task, with their relative contributions modulated by task conditions (e.g., distraction) (Poldrack and Rodriguez, 2004). Although a specific memory system may be governing a given task, redundant memory representations persist. For instance, deactivating the dorsal striatum can reactivate MTL-dependent response strategies, indicating that while declarative knowledge may not be utilized, it remains accessible (Packard and McGaugh, 1996).

It was determined in studies that cognitive depletion through a dual task of working memory and mental fatigue induction improved the ability to recognize words, especially under conditions where participants had less confidence in remembering words (e.g., when their knowledge was implicit). Inhibitory theta-burst stimulation (TBS) additionally modulated neural entrainment to the words and syllables (Smalle et al., 2022; Smalle et al., 2021). Cognitive depletion improves the acquisition of linguistic knowledge in adults by unlocking implicit statistical learning. Broca’s motor speech area, which is part of the premotor area of the brain, plays a role in speech production and motor control, and damage to this area leads to aphasia. In other words, the motor control of speech is limited to the hemisphere that controls the dominant upper motor organs (hand), concluding that there is a close relationship between the learning of verbal skills and the movements of the dominant hand (Bhuiyan et al., 2017). Moreover, many neuroimaging studies have also shown activation of Broca’s area in representing meaningful arm gestures. A study has shown that word and gesture are linked at the translation level of certain aspects of gesture, such as motor goal and intention (Gentilucci et al., 2006). Therefore, the similarities between the results of the present study and the works of Smalle et al. may be due to shared neural control centers between speech and dominant hand movements in the participants (corresponding to the research task, which was to throw the ball at the target with the dominant hand).

Based on the Optimal theory offered by Wulf and Lewthwaite (2016) enhanced expectancies, facilitated by successful experiences during practice, lead to improved goal-action coupling (Wulf and Lewthwaite, 2016). This process results in increased self-confidence, which subsequently reduces the internal focus of attention and enhances external goal-directed focus. Training conditions that foster greater successful experiences (such as errorless conditions) elicit dopaminergic responses by activating mesolimbic and nigrostriatal pathways (Deci and Ryan, 2008). The released dopamine strengthens and facilitates the neural connections essential for performance and learning, ultimately leading to more stable learning outcomes in individuals (Schultz, 2013; Schultz, 2010).

The lack of meaningful difference between mental and physical fatigue conditions suggests a complex relationship between these underlying mechanisms in central nervous system impairment. The similarity in results may be attributed to the multifaceted nature of fatigue and its impact on various neural regions. In most cases, acute physiological fatigue is accompanied by psychological stress. In response to psychological stressors, increased cortisol secretion and sympathetic activity (e.g., heart rate and blood pressure) have evolved to mobilize metabolic resources for a potential required action (Cannon, 1932). Also, releasing cortisol during physical activity to facilitate the metabolism of energy sources can reduce the ability of working memory (due to the reduction in ATP levels) (McArdle et al., 1996).

Elzinga and Roelofs (2005) suggested that this increase in cortisol levels, driven by sympathetic activity, is sufficient to cause impairments in working memory (Elzinga and Roelofs, 2005). Any impairment in the ability to use working memory could have disrupted motor output under the errorful (explicit) condition. It is entirely possible that the stress-induced elevation of cortisol, coupled with the marked increase in sympathetic activity resulting from our fatigue protocol, impaired the working memory capacity of our errorful group participants (Poolton et al., 2007).

With the onset of physical fatigue, an increase in voluntary effort is required to enhance motor output from the pre-motor cortex of the brain (Taylor and Gandevia, 2008). A physical facilitation system is responsible for this action, which comprises a neural circuit that interconnects the basal ganglia, thalamus, orbitofrontal cortex, dorsolateral prefrontal cortex, anterior cingulate cortex, pre-motor area, supplementary motor area, and primary motor cortex (Chaudhuri and Behan, 2004; Tanaka and Watanabe, 2012; Post et al., 2009; Liu et al., 2007; Korotkov et al., 2005; Chaudhuri and Behan, 2000). Conversely, there exists a mental facilitation system that fulfills the same function in response to mental fatigue. An increase in motivational inputs to this mental facilitation system, which includes the neural circuit connecting the basal ganglia, thalamus, and prefrontal cortex, is achieved by activating the dopaminergic system, aimed at compensating for mental fatigue (Chaudhuri and Behan, 2000; Lorist et al., 2009; Dobryakova et al., 2013).

In a study by Tanaka et al. (2016), it was shown that physical fatigue suppresses the oscillatory power of the alpha band in the left caudate (Tanaka et al., 2016). Due to the connection of this brain region with motivation, the reduction of alpha power is directly associated with an increase in subjective motivation levels. Therefore, the observed increase in reported subjective motivation during fatigue effectively compensates for the effects of mental fatigue.

Furthermore, a study on a motor task demonstrated that functional responses measured by near-infrared spectroscopy increased in the frontal lobe, and cognitive performance improved alongside this change (Yanagisawa et al., 2010). These results generally indicate that the cognitive facilitation system and the physical facilitation system share similar neural substrates. Physical fatigue impacts mental fatigue within the central nervous system, involving highly complex and interconnected underlying mechanisms.

The selected throwing task in this study and the method of inducing local fatigue closely resemble tasks such as free throw in basketball, archery, and goal shooting in water polo. Therefore, the results of this study may be generalizable to real-world performance contexts in these tasks.

In addition, the detrimental effects of fatigue - whether mental or physical - as an inseparable component of motor performance has been repeatedly demonstrated to affect various elements such as accuracy, technique, and motor coordination in different throwing tasks (Simsek et al., 2018; Daub et al., 2023; Royal et al., 2006). Mitigating these negative effects to prevent performance decline is a primary goal for trainers. According to the results of this study, selecting training methods such as errorless training creates more stable representations to cope with fatigued conditions and may even facilitate performance under fatigue.

In Wetzel (2018) study, a patient with post-stroke memory impairments achieved independence at discharge through an errorless learning strategy, requiring no physical assistance (Wetzel, 2018). Furthermore, in patients with moderate to severe Alzheimer’s disease, errorless training was shown to enhance cognitive rehabilitation exercises and is acknowledged as a valuable method in neurophysiological rehabilitation. These previous studies, aligned with the current findings, indicate that errorless training programs positively impact rehabilitation and performance enhancement (Śmigórska et al., 2019).

Overall, different motor activities can be associated with different forms of mental and physical fatigue. Also Patients with neurological and cognitive impairments often experience increased levels of exertion and fatigue, often due to sleep disturbances, muscle weakness, or central nervous system problems (Maestri et al., 2020). This fatigue can significantly reduce an individual’s performance potential. Therefore, strategic planning of exercise and training that incorporates implicit (errorless) methods can be beneficial in maintaining and even improving performance under conditions of fatigue.

## 6 Conclusion

To conclude our study’s results indicate that mental and physical fatigue improved the performance of the implicit learning group compared to their retention performance. These observations highlight the complex relationship between fatigue and motor functions and challenge the previous beliefs about the negative effects of fatigue on performance. The unexpected performance enhancement in the context of fatigue underscores the nuanced interplay between cognitive states, task demands, and learning outcomes in human performance. The present study’s limitations include the restricted number of exercise sessions and the lack of measurement of physiological factors such as heart rate and blood lactate levels, which could provide greater assurance of the physical fatigue protocol’s efficacy. In future research, we recommend expanding the number of learning sessions to assess the long-term effects of learning methods better. Additionally, detailed brain recordings are crucial for elucidating the intricate relationships within the memory system components. The authors also suggest that future studies should focus on different motor tasks or implement alternative fatigue induction protocols (such as aerobic fatigue, general fatigue, etc.) to clarify better the effects of implicit and explicit training under pressure conditions. This study provides valuable insights into optimizing motor skill learning methodologies, considering the interplay between explicit and implicit processes, especially under challenging conditions like mental and physical fatigue.

## Data Availability

The raw data supporting the conclusions of this article will be made available by the authors, without undue reservation.
